# Synthesis and Structural Characterization of the 5-(2-Haloethyl)pyrimidines – Hydrogen-Bonded chains in *α*-(1-Carbamyliminomethylene)-γ-Butyrolactone

**DOI:** 10.3390/molecules13112786

**Published:** 2008-11-06

**Authors:** Tatjana Gazivoda, Silvana Raić-Malić, Antonija Hergold-Brundić, Mario Cetina

**Affiliations:** 1 Department of Organic Chemistry, Faculty of Chemical Engineering and Technology, University of Zagreb, Marulićev trg 20, P.O. Box 177, HR-10000 Zagreb, Croatia; 2 Laboratory of General and Inorganic Chemistry, Faculty of Science, University of Zagreb, Horvatovac 102a, HR-10000 Zagreb, Croatia; 3 Department of Applied Chemistry, Faculty of Textile Technology, University of Zagreb, Prilaz baruna Filipovića 28a, HR-10000 Zagreb, Croatia

**Keywords:** 5-(2-Haloethyl)pyrimidine, Synthesis, X-ray crystal structure, Hydrogen bonds

## Abstract

Three novel 5-(2-haloethyl)pyrimidine derivatives were synthesized and characterized by ^1^H-NMR, ^13^C-NMR, MS, IR spectra and elemental analysis. Iodine and chlorine atoms in the C-5 side chain were introduced by reaction of 5-(2-hydroxyethyl)pyrimidine with hydroiodic acid and phosphoryl chloride, respectively. The structure of the intermediate α-(1-carbamyliminomethylene)-γ-butyrolactone was determined by X-ray crystal structure analysis. The molecule deviates very slightly from planarity. Three N−H···O hydrogen bonds link the molecules into one-dimensional chains of edge-fused rings.

## Introduction

**Scheme 1:**
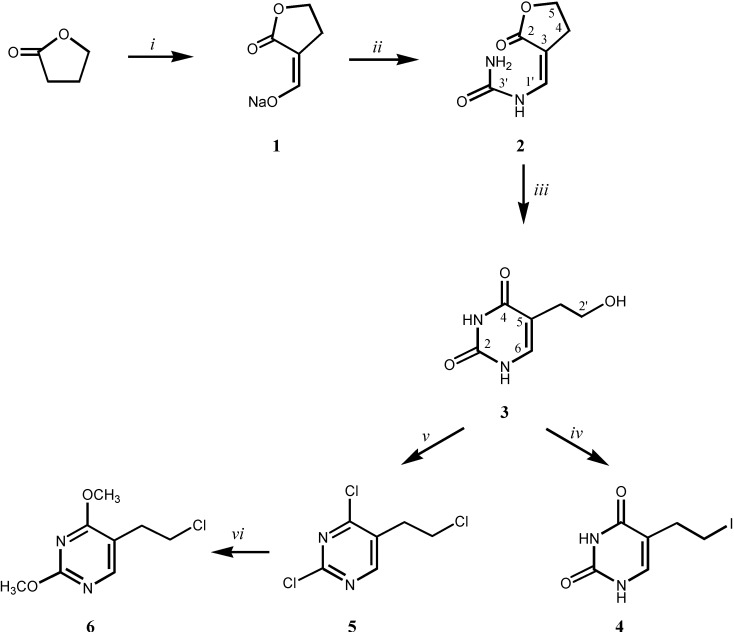
Synthesis of the 5-(2-haloethyl)pyrimidines **4**-**6**.

A number of 5-substituted pyrimidine nucleoside analogues have been shown to have potent and selective cytotoxic activity in cells transfected with the HSV-1 TK gene [1,2,3]. The (*E*)-5-(2-halovinyl)-2’-deoxyuridines are among the most potent and selective agents that undergo selective phosphorylation by the viral-encoded enzyme [4]. Pyrimidine nucleoside analogues, *e.g.* 5-(2-fluoroethyl)-2’-deoxyuridine, labelled with radioisotopes have applications in positron emission tomography (PET) to monitor the successful transfection of gene products *in vivo* during cancer-prodrug therapy [5]. Recently, we have reported that some C-5 and/or C-6 substituted pyrimidine derivatives exhibited cytostatic activities against human tumor cell lines [6,7,8,9,10,11]. In view of the importance of 5-substituted uracil derivatives in cancer chemotherapy [12] and as antiviral agents [13,14,15] we became interested in the synthesis of the novel 5-substituted uracils that we describe in this paper (Scheme 1). In addition, we also present herein the X-ray crystal structure of α-(1-carbamyliminomethylene)-γ-butyrolactone, a precursor in the synthesis of these 5-substituted uracils. This compound, possessing two hydrogen-bonding donor groups and two acceptors, is very interesting from the supramolecular point of view, because it can form intricate hydrogen-bonded networks including chain motifs, rings, chains of rings and chains of edge-fused rings.

*Reagents and conditions*: (*i*) NaOMe, ether, HCOOCH_3_, 24 h; (*ii*) urea, 3M HCl, 4 °C, 24 h; (iii) NaOEt, ethanol, reflux, 6 h; (*iv*) HI, -5 °C, 90 min; (*v*) POCl_3_, reflux, 1h; (*vi*) NaOMe, reflux, 6 h.

## Results and Discussion

### Synthesis

The sodium derivative **1**, prepared by reaction of γ-butyrolactone with methylformate in dry ether with the presence of sodium methoxide, was subsequently reacted with urea to give α-(1-carbamyliminomethylene)-γ-butyrolactone (**2**) [16,17]. Reaction of **2** with sodium ethoxide afforded 5-(2-hydroxyethyl)uracil (**3**), which with 57% solution of hydroiodic acid gave 5-(2-iodoethyl)uracil (**4**). Chlorination of compound **3** with phosphoryl chloride afforded the chlorinated pyrimidine derivative **5**, which was subsequently methoxylated giving 5-(2-chloroethyl)-2,4-dimethoxyuracil (**6**). The newly prepared compounds **4**-**6** were fully characterized by ^1^H- and ^13^C-NMR spectra, mass and IR spectra, as well as elemental analysis (see Experimental section).

### X-ray crystal structure study

**Figure 1:**
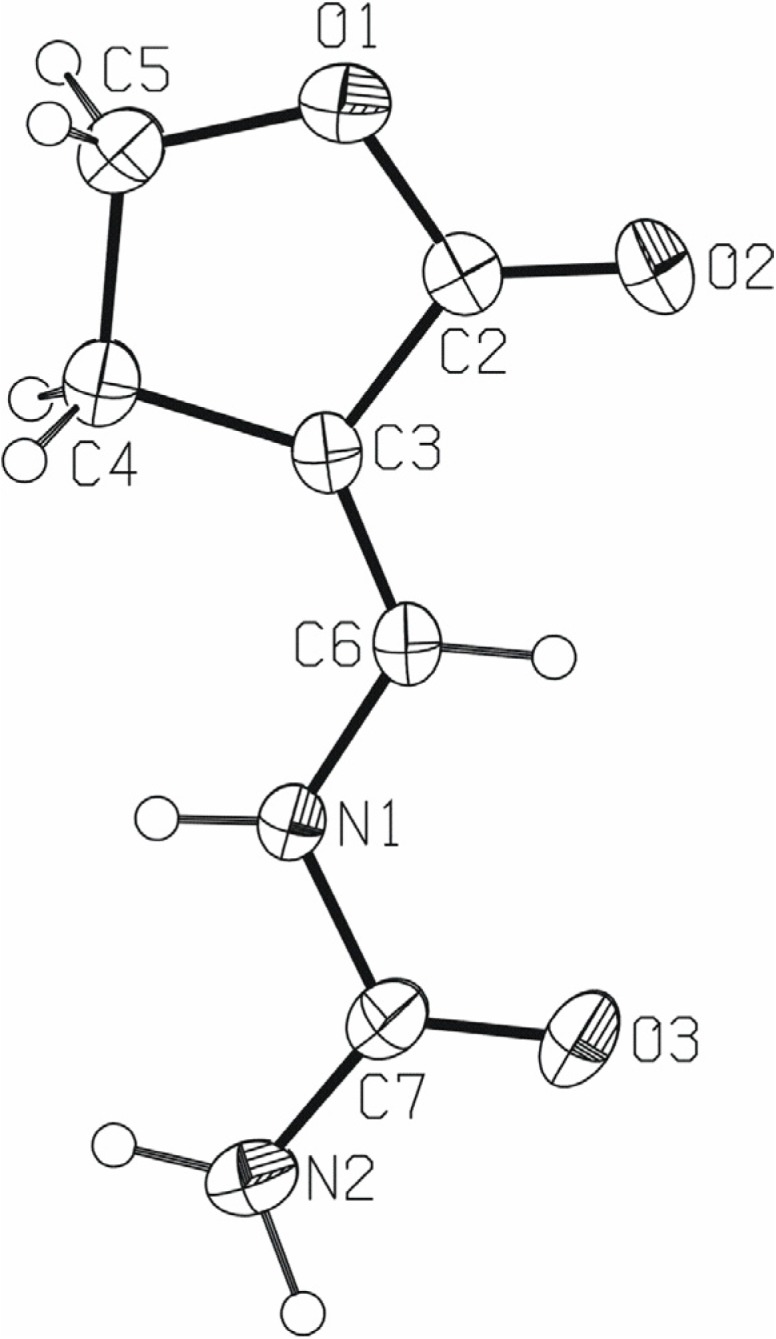
A molecular structure of **2**, with the atom-numbering scheme. Displacement ellipsoids for nonhydrogen atoms are drawn at the 40 % probability level.

Compound **2** (Figure 1) consists of a carbamyliminomethylene moiety bonded to the butyrolactone ring. The whole molecule deviates very slightly from planarity, as the dihedral angle between the mean planes of the carbamyliminomethylene atoms N2/C7/O3/N1/C6 and butyrolactone ring atoms O1/O2/C2/C3/C4/C5 is 7.17(8)^o^.

**Figure 2:**
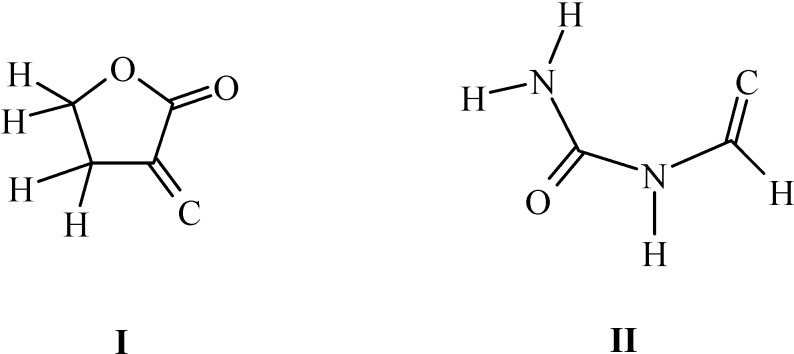
Fragments from a Cambridge Structural Database survey.

A survey of the Cambridge Structural Database [18] (CSD; error-free and disorder-free structures of organic compounds with R<0.075) revealed 16 structures in which the carbon atom is double bonded to the butyrolactone ring (Figure 2, fragment I) and only one structure possessing a carbamyliminomethylene moiety [19] (Figure 2, fragment II). 

The bond lengths and angles in **2** (Table 1) present no unexpected features and are within the ranges of equivalent ones in structures found in the CSD [18].

**Table 1:** Bond distances (Å) and bond angles (^o^) for **2**.

N1−C6	1.363(2)		O3−C7	1.2251(19)
N1−C7	1.3900(18)		C2−C3	1.453(2)
N2−C7	1.339(2)		C3−C6	1.333(2)
O1−C2	1.3417(17)		C3−C4	1.495(2)
O1−C5	1.450(2)		C4−C5	1.518(2)
O2−C2	1.2173(19)			
C6−N1−C7	121.43(13)		C2−C3−C4	108.73(12)
C2−O1−C5	110.77(12)		C3−C4−C5	102.74(13)
O2−C2−O1	120.86(14)		O1−C5−C4	107.73(12)
O2−C2−C3	129.10(15)		C3−C6−N1	125.09(13)
O1−C2−C3	110.02(12)		O3−C7−N2	124.88(15)
C6−C3−C2	121.18(13)		O3−C7−N1	121.05(15)
C6−C3−C4	130.00(14)		N2−C7−N1	114.07(13)

**Table 2:** Hydrogen-bonding geometry for **2**.

D−H···A	D−H (Å)	H···A (Å)	D···A (Å)	D−H··· A
N1−H1···O2*^i^*	0.85(2)	2.06(2)	2.869(2)	158(2)
N2−H2A···O2*^i^*	0.90(2)	2.16(2)	2.974(2)	150(2)
N2−H2B···O3*^ii^*	0.93(2)	1.96(2)	2.884(2)	174(2)

Symmetry codes: (*i*) x, −1+y, z; (*ii*) 1−x, −1/2+y, 3/2−z.

The carbamyliminomethylene moiety nitrogen atoms N1 and N2 and carbonyl oxygen atoms O2 and O3 participate in the supramolecular assembly of **2**. The N2 atom is a double proton donor and O2 atom is a double proton acceptor (Table 2).

**Figure 3:**
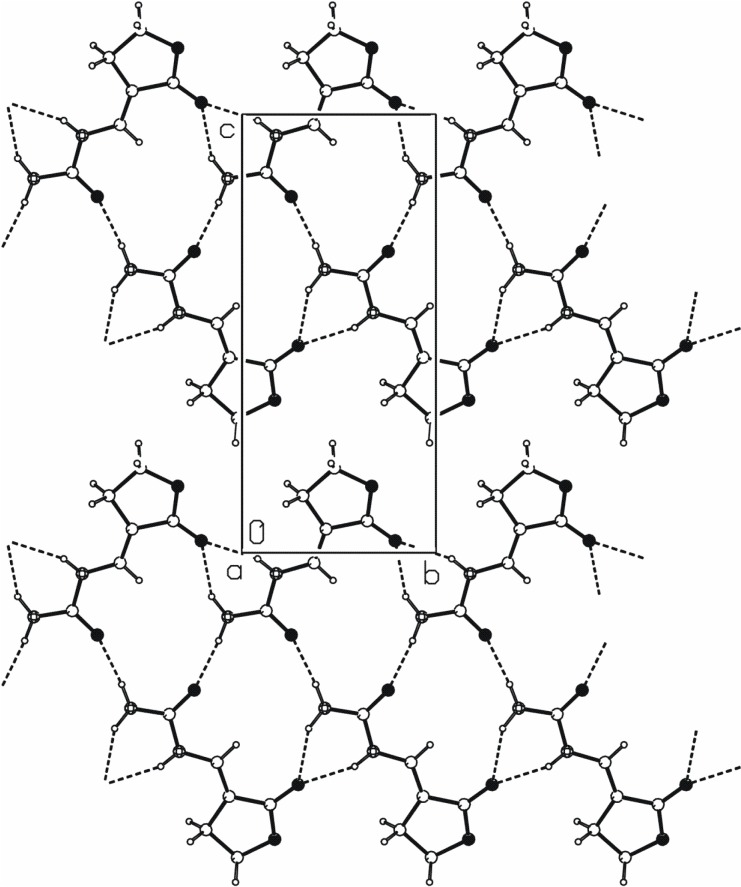
A crystal packing diagram of **2**, viewed along the *a* axis, showing chains of edge-fused rings formed by N−H···O hydrogen bonds.

The N1···O2*^i^* and N2···O2*^i^* intermolecular hydrogen bonds in **2** form a 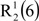 [20] chain of rings parallel to the *b* axis (Figure 3). The N2···O3*^ii^* hydrogen bond links the molecules into *C*(4) chains that are also parallel to the *b* axis. The combination of these two motifs forms new ring of 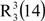 type. These three N−H···O hydrogen bonds generate mutually parallel one-dimensional chains of edge-fused rings (Figure 3).

**Figure 4:**
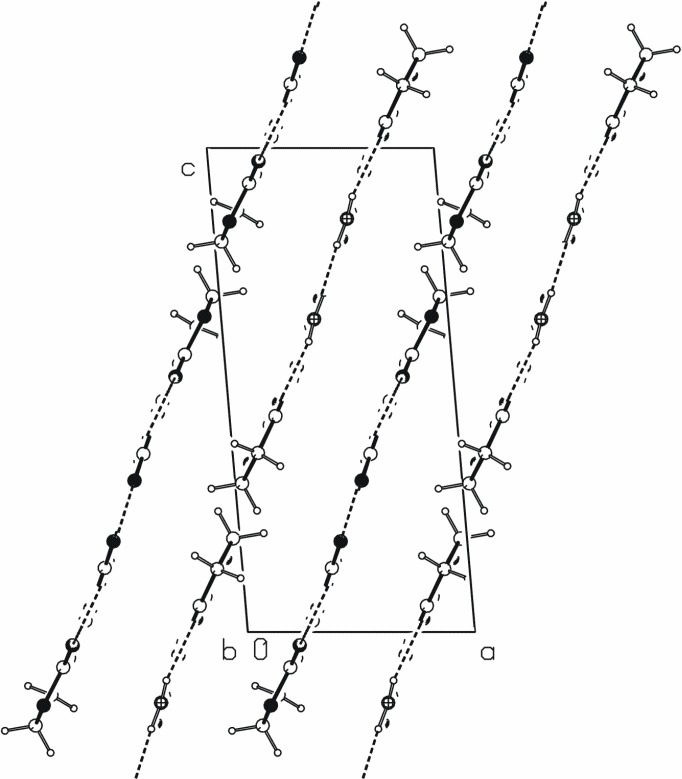
A crystal packing diagram of **2**, viewed along the *b* axis, showing parallel chains of hydrogen-bonding molecules.

The crystal packing diagram along *b* axis (Figure 4) reveals that the hydrogen-bonded chains are parallel to the (2, 0, 1). The distance between the planes passing through the center of the hydrogen-bonded molecules is approximately 3.05 Å. Very similar one-dimensional hydrogen-bonded chains generate N−H···O hydrogen bonds in 1-carbamyliminomethylenetetrahydropyrrol-2-one [19].

## Experimental Section

### General

Melting points were determined on a Kofler micro hot-stage apparatus (Reichert, Wien) and are uncorrected. The electron impact mass spectra were recorded with an EXTREL FT MS 2002 instrument with ionizing energy of 70 eV. ^1^H- and ^13^C-NMR spectra were recorded on a Bruker Avance 600 MHz spectrometer, operating at 600 MHz (^1^H) and 150.92 MHz (^13^C), respectively. The samples were dissolved in DMSO-*d*_6_ and measured in 5 mm NMR tubes. The ^1^H- and ^13^C-NMR chemical shift values (δ) are expressed in ppm referred to tetramethylsilane (TMS) and coupling constants (*J*) in Hz. The infrared spectra were recorded by a Fourier transform infrared (FTIR) spectrometer Bruker Vertex 70 equipped with an attenuated total reflection (ATR) accessory with a diamond crystal. Sixteen scans were collected for each measurement over the spectral range of 400-4000 cm^-1^ with resolution of 4 cm^-1^. Precoated Merck Silica Gel 60F-254 plates were used for thin layer chromatography (TLC) and the spots were detected under UV light (254 nm). Column chromatography (CLC) was performed using silica gel (0.063-0.2 mm) Fluka; glass column was slurry-packed under gravity. Compound purity was analyzed by HPLC with DAD detector. 

### Synthesis

*Sodium α-hydroxymethylene-γ-butyrolactone* (**1**) [16]. To a cooled suspension of NaOMe (35.5 g, 0.66 mol) in dry ether (650 mL), a solution of methylformate (52.6 mL, 0.85 mol) and γ-butyrolactone (50 mL, 0.66 mol) was added dropwise. The stirring was continued at room temperature overnight. After cooling of reaction mixture the precipitated product **1** was filtered off and washed with dry ether (37.5 g, 41.8 %, m.p. = 124 – 126 °C); MS *m/z* (EI) for C_5_H_5_NaO_3_ (M^+^^•^) calc. 136.0812; found 136.1; ^1^H-NMR δ 8.46 (1H, s, H-1'), 4.24 (2H, t, *J* = 7.00 Hz, H-5), 2.44 (2H, dt, *J* = 9.97 Hz, 8 Hz, H-4); ^13^C-NMR δ 176.96 (C-2), 166.54 (C-1'), 132.60 (C-3), 68.37 (C-5), 27.50 (C-4).

*α-(1-Carbamyliminomethylene)-γ-butyrolactone* (**2**) [17]. To a solution of urea (30 g, 0.5 mol) in cold 3M HCl (200 mL) was added sodium α-hydroxymethylene-γ-butyrolactone (**1**, 34 g, 0.25 mol). After stirring at 4 °C overnight the precipitated product was filtered off and washed with cold water. Recrystallization from water – ethanol gave white crystals of **2** (2 g, 5.2 %, m.p. = 246 – 247 °C); MS *m/z* (EI) for C_6_H_8_N_2_O_3_ (M^+^^•^) calc. 156.1394; found 156.1; ^1^H NMR δ 8.96 (1H, d, *J* = 11.71 Hz, NH), 7.60 (1H, d, *J* = 11.97 Hz, H-1'), 6.38 (2H, s, NH_2_), 4.28 (2H, t, *J* = 7.5 Hz, H-5), 2.75 (2H, dt, *J* = 7.5 Hz, 2.3 Hz, H-4); ^13^C-NMR δ 172.69 (C-3'), 154.35 (C-2), 132.60 (C-1'), 99.30 (C-3), 65.07 (C-5), 24.19 (C-4).

*5-(2-Hydroxyethyl)uracil* (**3**). To a solution of NaOEt (0.277 g, 4 mmol) in ethanol (20 mL) was added α-(1-carbamyliminomethylene)-γ-butyrolactone (**2**, 1.7 g, 0.01 mol). Reaction mixture was refluxed for 6 h, during which time product precipitated. The solvent was removed under reduced pressure and the residue was dissolved in the water and acidified to pH = 3. The precipitated product **3** was filtered off, washed with ethanol and dried *in vaccuo* (1.55 g, 91.2 %); MS *m/z* (EI) for C_6_H_8_N_2_O_3_ (M^+^^•^) calc. 156.1394; found 156.1; ^1^H-NMR δ 11.41 (1H, s, NH), 11.13 (1H, s, NH), 7.21 (1H, s, H-6), 3.63 (1H, t, *J* = 6.68 Hz, OH), 3.41 (2H, t, *J* = 6.62 Hz, H-2'), 2.29 (2H, t, *J* = 6.51 Hz, H-1'); ^13^C-NMR δ 164.32 (C-4), 151.50 (C-2), 135.19 (C-6), 110.38 (C-5), 60.21 (C-2'), 29.06 (C-1').

*5-(2-Iodoethyl)uracil* (**4**). To a cooled solution of **3** (100 mg, 0.6 mmol) in H_2_O (10 mL), a solution of HI (57%, 1 mL) was added. The reaction mixture was stirred at -5 °C for 90 min, then solvent was evaporated and residue was purified by column chromatography (CH_2_Cl_2_:CH_3_OH = 60:1) to give oily product **4** (112 mg, 65 %); MS *m/z* (EI) for C_6_H_7_IN_2_O_2_ (M^+^^•^) calc. 266.0365; found 266; Anal. for C_6_H_7_IN_2_O_2_ calc. C 27.09 %, H 2.65 %, N 10.53 %; found C 27.13 %, H 2.64 %, N 10.55 %; IR: 3110–2992 (ν N–H, CH_2_), 1720 (ν C=O), 1650–1420 (ν C=C and C–H ring), 1480 (δ CH_2_), 1022–770 (δ C=C–H, CH_2_), 940 (δ CH_2_–I), 520–480 (ν C–I) cm^-1^; ^1^H-NMR δ 10.97 (1H, s, NH), 10.62 (1H, s, NH), 7.20 (1H, s, H-6), 4.52 (2H, t, *J* = 5.4 Hz, H-2'), 2.28 (2H, t, *J* = 6.60 Hz, H-1'); ^13^C-NMR δ 165.14 (C-4), 151.82 (C-2), 139.27 (C-6), 109.61 (C-5), 59.86 (C-2'), 20.03 (C-1').

*5-(2-Chloroethyl)-2,4-dichloropyrimidine* (**5**). The reaction mixture of **3** (600 mg, 4 mmol) and POCl_3_ (17.7 mL, 0.19 mol) was refluxed for 1 h. Solvent was evaporated and ice was added in the residue which was then washed with ether and dried over Na_2_SO_4_. After drying the solvent was removed *in vacuo* and residue was purified by column chromatography (CH_2_Cl_2_:CH_3_OH = 30:1) which gave colourless oily product **5** (260 mg, 31%); MS *m/z* (EI) for C_6_H_5_Cl_3_N_2_ (M^+^^•^) calc. 211.4575; found 211.5; Anal. for C_6_H_5_Cl_3_N_2_ calc. C 34.08 %, H 2.38 %, N 13.25 %; found C 34.01 %, H 2.39 %, N 13.22 %; IR: 1633–1320 (ν C=C and C–H ring), 1430 (δ_s_ CH_2_), 1090 (δ CH_2_–Cl), 980-760 (δ C=C–H, CH_2_), 580–510 (ν C–Cl) cm^-1^; ^1^H-NMR δ 8.82 (1H, s, H-6), 3.93 (2H, t, *J* = 6.78 Hz, H-2'), 3.19 (2H, t, *J* = 6.78, H-1'); ^13^C-NMR δ 172.46 (C-4), 162.01 (C-2), 156.87 (C-6), 112.70 (C-5), 43.15 (C-2'), 31.51 (C-1').

*5-(2-Chloroethyl)-2,4-dimethoxypyrimidine* (**6**). To a solution of NaOMe (120 mg, 2.2 mmol) in MeOH (15 mL) was added **5** (80 mg, 0.38 mmol), and the mixture was heated at reflux for 6 h. The solvent was evaporated, and H_2_O was added to dissolve NaCl. The oily layer was extracted with CH_2_Cl_2_, dried (Na_2_SO_4_), and concentrated under reduced pressure. The residue was purified by column chromatography (CH_2_Cl_2_:CH_3_OH = 30:1) which gave colourless oily product **6** (54 mg, 70.6 %); MS *m/z* (EI) for C_8_H_11_ClN_2_O_2_ (M^+^^•^) calc. 202.6379; found 202.6; Anal. C 7.42 %, H 5.47 %, N 13.82 %; found C 47.31 %, H 5.47 %, N 13.84 %; IR: 2960 (ν CH_3_), 1640–1330 (ν C=C and C–H ring), 1420 (δ CH_2_), 1210 (ν_as_ C–O–C), 1100 (δ CH_2_–Cl), 1050 (ν_s_ C–O–C), 905–760 (δ C=C–H, CH_2_), 630–520 (ν C–Cl) cm^-1^; ^1^H-NMR δ 8.19 (1H, s, H-6), 3.92 (3H, s, OCH_3_), 3.87 (3H, s, OCH_3_), 3.77 (2H, t, *J* = 6.93 Hz, H-2'), 2.89 (2H, t, *J* = 6.93 Hz, H-1'); ^13^C-NMR δ 169.30 (C-4), 164.56 (C-2), 158.87 (C-6), 111.44 (C-5), 54.85 (OCH_3_), 54.33 (OCH_3_), 43.75 (C-2'), 29.76 (C-1').

### Crystal structure determination of **2**

A single crystal of **2** suitable for X-ray structure analysis was prepared by growth under slow evaporation at room temperature of a very dilute ethanol solution (v/v 96 %). The intensities were collected at 295 K on a Philips PW1100 diffractometer updated by Stoe and Cie [21,22] using Mo-K_α_ radiation (0.71073 Å). Details of crystal data, data collection and refinement parameters are given in Table 3.

**Table 3:** X-ray crystallographic data for **2**.

Formula	C_6_H_8_N_2_O_3_
Formula weight	156.14
Crystal system	monoclinic
Space group	*P* 2_1_/c
Unit cell dimensions	
*a* (Å)	7.0522(8)
*b* (Å)	6.6691(6)
*c* (Å)	15.1031(16)
*β* (^o^)	94.869(9)
*V* (Å^3^)	707.76(13)
*Z*	4
*D*_calc._ (g cm^-3^)	1.465
Absorption coefficient *μ* (mm^-1^)	0.119
*F*(000)	328
*θ* range (°)	2.71 - 27.99
Index ranges	-9 ≤ h ≤ 9
	-8 ≤ k ≤ 8
	-11 ≤ l ≤ 19
Collected reflections No.	2941
Independent reflections No. / *R*_int._	1702 / 0.0231
Reflections No. *I* ≥ 2*σ*(*I*)	1063
Refined parameters No.	112
Goodness-of-fit on F^2^, *S*	1.001
*R* [I ≥ 2σ(I)] / *R* [all data]	0.0398 / 0.0783
w*R* [I ≥ 2σ(I)] / w*R* [all data]	0.1061 / 0.1231
Max./min. electron density (e Å^-3^)	0.189 / -0.141

The structure was solved by direct methods [23]. All non-hydrogen atoms were refined anisotropically by full-matrix least-squares calculations based on F^2^ [23]. The hydrogen atoms bonded to nitrogen atoms were found in a difference Fourier map and their coordinates and isotropic thermal parameters have been refined freely. All other hydrogen atoms were included in calculated positions as riding atoms, with SHELXL97 [23] defaults. PLATON [24] program was used for structure analysis and molecular and crystal structure drawings preparation. CCDC 695308 contains the supplementary crystallographic data for this paper. These data can be obtained free of charge via www.ccdc.cam.ac.uk/data_request/cif, by emailing data_request@ccdc.cam.ac.uk, or by contacting The Cambridge Crystallographic Data Centre, 12, Union Road, Cambridge CB2 1EZ, UK; fax: +44 1223 336033.
